# STAT Is an Essential Activator of the Zygotic Genome in the Early *Drosophila* Embryo

**DOI:** 10.1371/journal.pgen.1002086

**Published:** 2011-05-26

**Authors:** Amy Tsurumi, Fan Xia, Jinghong Li, Kimberly Larson, Russell LaFrance, Willis X. Li

**Affiliations:** 1 Department of Biomedical Genetics, University of Rochester Medical Center, Rochester, New York, United States of America; 2 Department of Medicine, University of California San Diego, La Jolla, California, United States of America; University of California San Francisco, United States of America

## Abstract

In many organisms, transcription of the zygotic genome begins during the maternal-to-zygotic transition (MZT), which is characterized by a dramatic increase in global transcriptional activities and coincides with embryonic stem cell differentiation. In *Drosophila*, it has been shown that maternal morphogen gradients and ubiquitously distributed general transcription factors may cooperate to upregulate zygotic genes that are essential for pattern formation in the early embryo. Here, we show that *Drosophila* STAT (STAT92E) functions as a general transcription factor that, together with the transcription factor Zelda, induces transcription of a large number of early-transcribed zygotic genes during the MZT. STAT92E is present in the early embryo as a maternal product and is active around the MZT. DNA–binding motifs for STAT and Zelda are highly enriched in promoters of early zygotic genes but not in housekeeping genes. Loss of *Stat92E* in the early embryo, similarly to loss of *zelda*, preferentially down-regulates early zygotic genes important for pattern formation. We further show that STAT92E and Zelda synergistically regulate transcription. We conclude that STAT92E, in conjunction with Zelda, plays an important role in transcription of the zygotic genome at the onset of embryonic development.

## Author Summary

In the initial phase of the early embryo, transcription is inactive and development is supported by maternally derived gene products. During a time window termed the maternal-to-zygotic transition (MZT), the maternal gene products are degraded and the zygotically expressed genes required for embryogenesis initiate their transcription. How the dramatic upregulation of zygotic genes during the MZT is achieved is not completely understood, although it has been shown that the transcription factor Zelda plays a critical role. In this manuscript, we show that *Drosophila* STAT (STAT92E) functions as a general transcription factor that, together with Zelda, induces transcription of a large number of early-transcribed zygotic genes during the MZT. We further show that STAT92E and Zelda synergistically regulate transcription. Thus, multiple transcription factors, such as STAT92E and Zelda, cooperate to control transcription of the zygotic genome at the onset of embryonic development.

## Introduction

Embryonic pattern formation is a complex and progressive process. In many multicellular organisms, the initial period of embryogenesis relies on gene products inherited from the mother. In *Drosophila*, maternally derived morphogen proteins form broad gradients along the major body axes to define body polarities [1]–[3]. Zygotic transcription begins during the maternal-to-zygotic transition (MZT), which is characterized by a decline in maternal mRNA levels and a dramatic increase in a large number of zygotic transcripts [4], [5]. Many of the zygotic genes transcribed the earliest, exhibit region-specific patterns. For instance, the “gap genes”, such as zygotic *hunchback* (*hb*), *Krüppel (Kr), knirps (kni)*, and *tailless (tll)* are transcribed zygotically in broad and mostly non-overlapping domains along the anteroposterior (A/P) body axis. The boundaries of these zygotic genes are determined by morphogen gradients that are set up by maternal gene products, such as Bicoid (Bcd) and maternal Hb [2], [3]. Additional zygotic genes, mostly transcription factors, are induced in more refined embryonic regions as a result of cooperation between the maternal morphogens and gap gene products. The combinatorial input of different transcription factors at different positional coordinates results in expression of thousands of zygotic genes in an increasingly refined pattern, leading to cell fate determination and differentiation [1]–[3], [6].

To date, only a few transcription factors have been implicated in transcription of the zygotic genome during the MZT. For example, the maternal morphogens Bcd and Dorsal activate target genes along the anteroposterior (A/P) and dorsoventral (D/V) axis, respectively [7], [8]. The dramatic increase in gene expression that occurs during the MZT raises the possibility that additional unidentified transcription factors are involved in the rapid initiation and maintenance of the heightened levels of zygotic gene transcription that characterize the MZT. It has been proposed that the few known regionally localized transcription factors, such as Bcd and Dorsal, act in conjunction with ubiquitously present factors to induce and maintain expression of a large number of zygotic genes in cell type-specific patterns. This idea is supported by the identification of a ubiquitous factor encoded by *zelda* (*zld*; a.k.a. *vielfaltig* or *vlf*) [9], and further by the demonstration that combining Dorsal with Zelda- or STAT-binding sites supports transcription in a broad domain in the embryo [10].

To identify additional ubiquitous transcription factors that are important for transcription of the zygotic genome during the MZT, we first conducted *in silico* analyses, taking advantage of the large amount of information available in public databases on transcriptional regulation of zygotic genes expressed during early embryogenesis in *Drosophila*. This approach led to the identification of STAT92E, in addition to Zelda, as a plausible transcription factor important for the upregulation of multiple genes during the MZT. Global expression profiling studies indicate that loss of STAT92E, similarly to loss of Zelda, preferentially causes down-regulation of zygotic genes essential for early embryogenesis. We further demonstrate that STAT92E is indeed involved in transcription of the developmentally important genes *dpp, tailless* (*tll*), and *Kr* during early embryogenesis. Our results suggest that STAT92E is essential for upregulation of a multitude of zygotically transcribed genes during the MZT, and thus is important for transition of the early embryo from a totipotent embryonic stem cell state to a state of cellular differentiation.

## Results

### *In silico* identification of factors important for transcription of the zygotic genome

To identify general transcription factors that are required for transcription of a large number of zygotic genes at early embryonic stages, or during the MZT, we performed a meta-analysis to search for candidate transcription factors required for activation of multiple zygotic genes. To this end, we first selected a list of developmentally important zygotic genes transcribed during the MZT (referred to as “zygotic genes”), whose expression patterns altogether cover the entire embryo, and whose transcriptional activation has previously been studied. We analyzed a total of 21 early zygotic genes, including the gap genes: *hunchback* (*hb*), *huckebein (hkb), Giant (Gt), Krüppel (Kr), knirps (kni),* and *tailless (tll)*; the pair-rule genes: *even skipped (eve), fushi tarazu (ftz), hairy (h), odd paired (opa), paired (prd), sloppy paired 1 (slp1),* and *runt (run)*; the segmental polarity and other genes: *engrailed (en)* and *Sex lethal* (*Sxl*), as well as genes expressed along the D/V axis: *decapentaplegic (dpp), zerknüllt (zen), rhomboid (rho), short gastrulation (sog), snail (sna),* and *twist (twi*).

**Figure 1:**
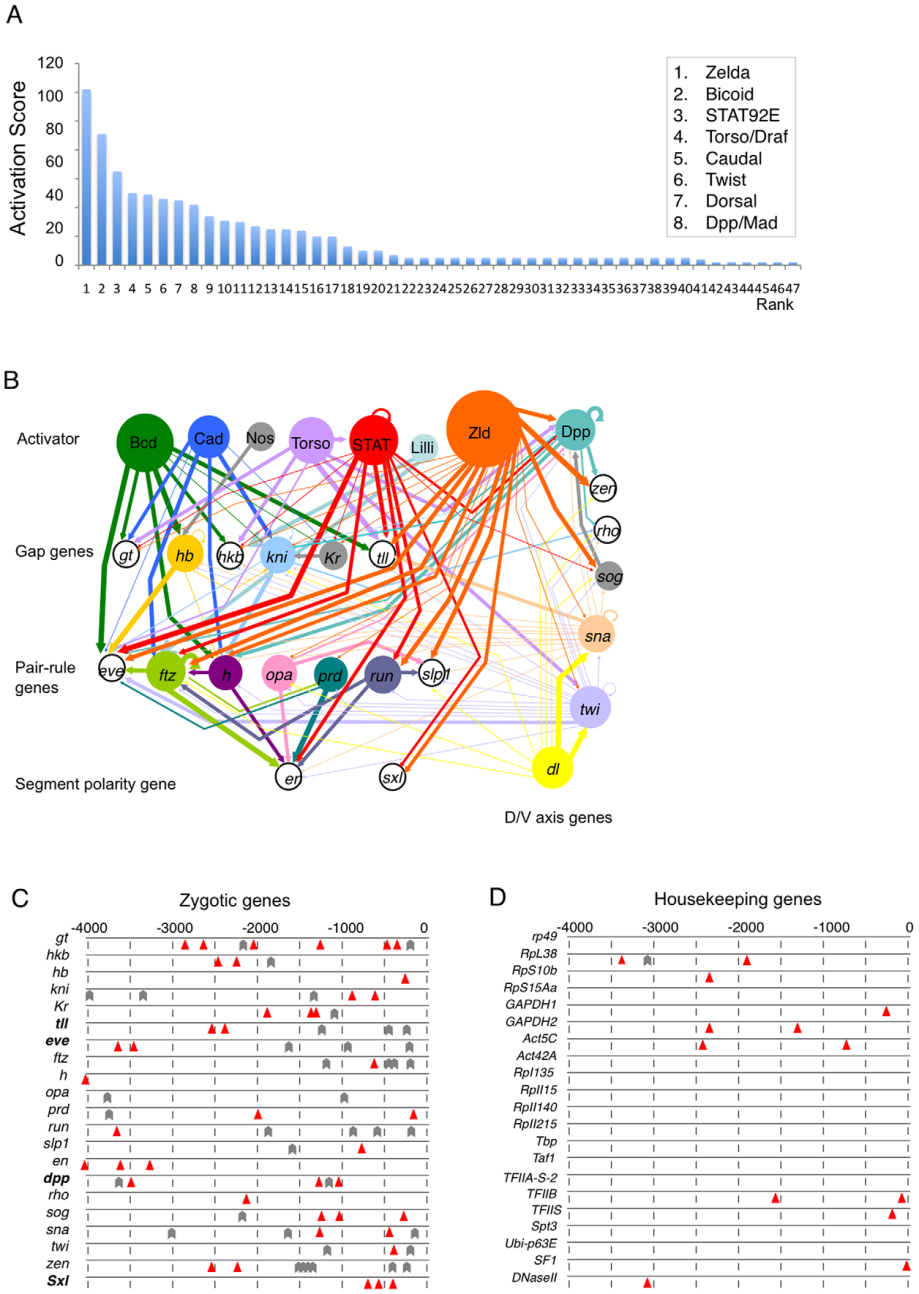
Factors contributing to zygotic gene expression during the MZT. (A) Activation scores for transcription factors or signaling pathways important for transcriptional upregulation of a set of 21 zygotically expressed genes. The top eight factors are indicated. See Table S2 for a complete list of factors and their scores. (B) An activation map showing connections between activators (top row) and their target genes, grouped as gap genes, pair-rule genes, segment polarity genes, and genes expressed along the D/V axis. Lines indicate activation (some are indirect). The thickness of the line represents the activation strength based on meta-analysis. (C, D) Horizontal lines represent promoter regions of the indicated early zygotic genes (C) or housekeeping genes (D). Numbers indicate base pairs upstream (–) of the transcriptional start site (0). Red triangles represent consensus STAT92E binding sites (TTCnnnGAA). Gray arrowheads indicate the positions of Zelda-binding consensus sequences (CAGGTAG). Bold gene names indicate the promoter regions as shown are known to support expression. A list of additional housekeeping genes can be found in Table S3.

As a second step, for each of these genes, we searched Flybase (http://flybase.org) and PubMed (http://www.ncbi.nlm.nih.gov), and compiled a list of all currently known or potential transcriptional activators or signaling pathways involved in their transcriptional induction (Table S1). We used the RedFly database (http://redfly.ccr.buffalo.edu) [11] to obtain a list of experimentally verified transcription factor binding sites for each target gene, and the FlyEnhancer program (http://genomeenhancer.org/fly) [12] to search for the presence of particular transcription factor binding sites in the promoter region (defined as 4 kb upstream of the transcriptional start site) of all the target genes. Based on these search results, we assigned activation scores to the putative or known transcriptional activators to reflect their importance in the expression of a particular zygotic gene (Table S1). These scores were added to obtain a cumulative score for each activator (Figure 1A; Table S2). The connections between activators and their target genes are represented in an activation map (Figure 1B).

The top seven activators identified, in descending order of cumulative interaction score, were Zelda (Zld), Bicoid (Bcd), STAT92E, Torso, Caudal (Cad), Dorsal, and Twist (Twi) (Figure 1A; Table S2). Zelda has previously been shown to be a key transcription activator of the early zygotic genome [9], validating our bioinformatic approach. Both Bcd and Cad are maternal-effect gene products that form gradients along the A/P axis in the early embryo [7], [13], [14]; Torso signaling is activated only at the anterior and posterior poles, and the specific transcriptional activators that it regulates remain unidentified [15]–[17]; Dorsal and Twi are active only in the ventral region of the embryo [18]. On the other hand, STAT92E is ubiquitously distributed in the early embryo as a maternal product [19] and is activated early [20], and thus has the potential to act more universally. STAT92E is the transcriptional activator mediating the JAK/STAT (Hop/STAT92E) pathway [19], [21], [22], and also participates in Torso signaling [23]–[25]. Thus, we decided to investigate whether STAT92E acts as a general transcriptional regulator during early embryogenesis, similar to Zelda.

### STAT- and Zelda-binding sites are enriched in promoter regions of early zygotic genes

To test whether STAT92E is important for transcription of early “zygotic genes”, we first assessed the occurrence of consensus STAT92E binding sites (TTCnnnGAA) in the promoter region, defined as 4 kb genomic sequence upstream of the transcription start site, of the 21 zygotic genes in this study. The *Drosophila* genome is slightly AT-rich, with 57.4% AT and 42.6% GC base pairs [12]. Thus the probability for A or T to occur at any position is 0.287, and for G or C is 0.213, and the probability (*p*) for random occurrence of one STAT binding site (with 6 fixed nucleotides) at any position is 3.08x10−4 (0.2874x0.2132), and its frequency of occurrence within the 4 kb upstream regulatory regions of 21 genes (n = 84,000 bp) at random is 25.9 (*np*; expected value). However, when we searched for STAT binding sites within the 4 kb upstream region of the 21 zygotic genes, we found 43 in total (observed value) (Figure 1C). Assuming the actual occurrence of STAT-binding sites exhibits Binomial distribution with a probability of 3.08x10−4, the standard deviation (σ) should be 5.1. The difference between the observed (43) and expected (25.9) values is 17.1, which is beyond three standard deviations (Z = 3.29; *p* = 0.001).

In contrast, when we searched for STAT-binding sites within a 4 kb window upstream of the transcription start site of 21 housekeeping genes (defined as ubiquitously expressed, both maternally and zygotically, with generally cellular metabolic or structural functions), including *rp49, GAPDH, Actin5C*, and those encoding ribosomal proteins and RNA polymerases, we found a total of 13 STAT-binding sites (Figure 1D), which is significantly lower than the expected 25.9 sites (Z = 2.48; p = 0.013). (A total of 78 housekeeping genes and the numbers of STAT-binding sites in their upstream regions are listed in Table S3.) Moreover, many of the STAT-binding sites in the upstream regions of the 21 zygotic genes are clustered (defined by two sites occurring within 500 bp), which is characteristic of functional transcription factor binding sequences [12], [19], [25], [26] (Figure 1C), whereas in the promoter regions of the 21 housekeeping genes, the STAT-binding sites occur as single sites (Figure 1D; Table S3).

It has been shown that Zelda-binding sites (the TAGteam motif) are enriched in the promoter regions of “zygotic genes” [9], [27]. We examined the distribution of Zelda-binding sites in the promoter regions of the 21 zygotic and housekeeping genes, respectively. Consistent with the previous report [9], [27] and similar to STAT-binding sites, we found that Zelda-binding sites are similarly enriched in the promoters of the zygotic and very infrequently in the housekeeping genes (Figure 1C, 1D). Since the enhancers for many of the early zygotic genes are not localized in the upstream promoter regions, we also searched for STAT and Zelda-binding sites in the promoter-distal enhancers for these 21 zygotic genes, and found that promoter-distal enhancers are not enriched for STAT-binding sites (Z = 0.63; p = 0.736), but are significantly enriched for Zelda-binding sites (Z = 3.13; p = 0.0017) (Figure S1). Such a result suggests that STAT92E might differ from Zelda and might not be important for regulating promoter-distal enhancers, which usually control spatial expression patterns. Nonetheless, our studies indicate that DNA-binding sites for both STAT and Zelda are enriched in the upstream promoter regions of the 21 zygotic genes that are highly transcribed during the MZT, but are underrepresented in the housekeeping genes that are ubiquitously transcribed. This observation is consistent with the finding that Zelda is required specifically for expression of “zygotic genes” at the MZT [9], raising the possibility that STAT may play a similar role.

### Similar to Zelda, STAT92E is required for transcription of the zygotic genome during the MZT

To determine whether STAT92E functions as a general transcriptional activator of the zygotically expressed genes in the early embryo, we determined the expression profiles of early stage embryos (corresponding to nuclear division cycle 8–14, a time window for the MZT) of wild-type control and of those lacking the maternal *Stat92E* gene products (referred to as *Stat92E^mat–^*; see Methods) at the same stage.

**Figure 2:**
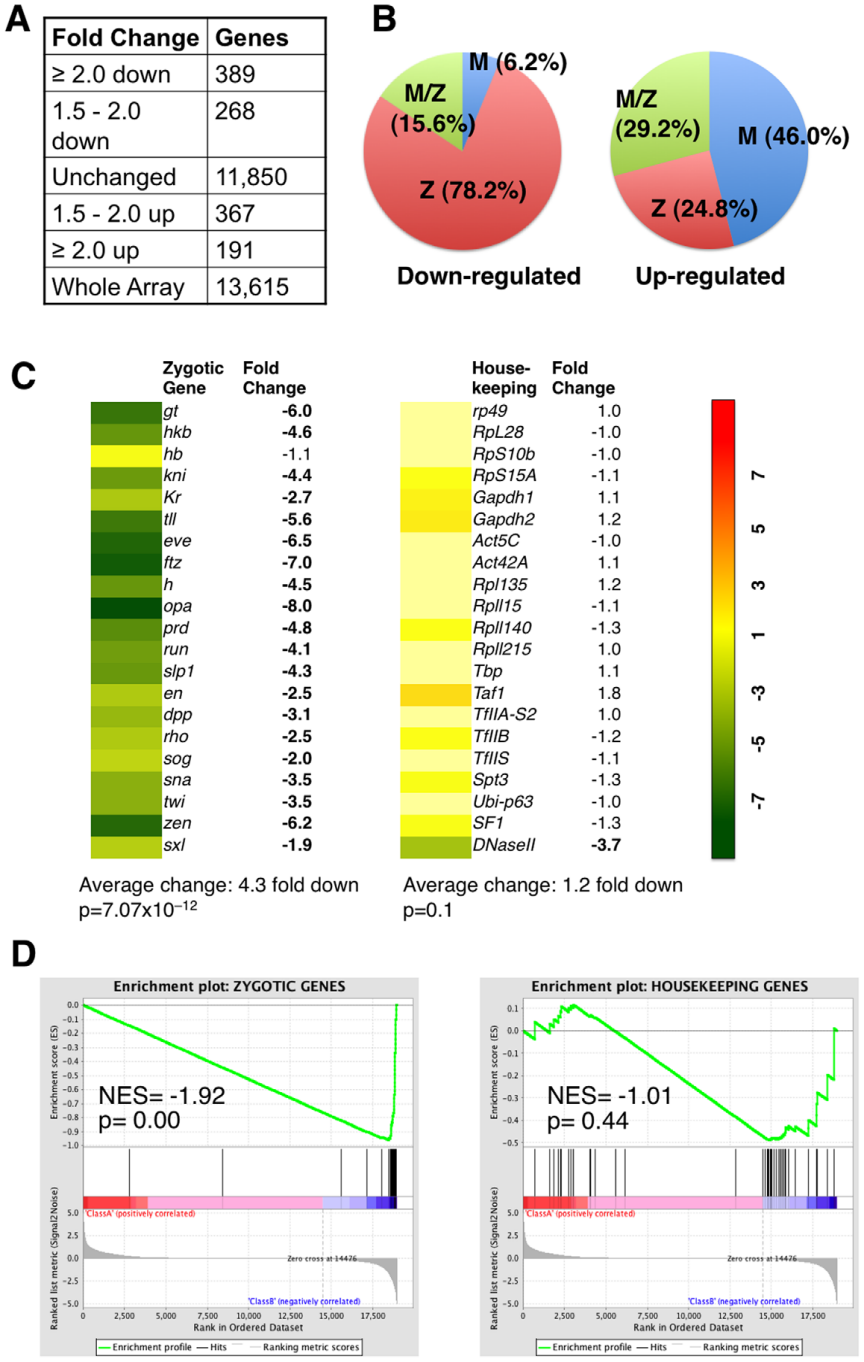
Expression profiles of embryos lacking maternal STAT92E. RNA isolated from 1–2 h wild-type and *Stat92E^mat–^* embryos were subjected to microarray analysis. (A) Summary of expression profiles of *Stat92E^mat–^* versus wild-type embryos. (B) Percent of genes categorized as zygotic (Z), maternal (M), or both (M/Z) in the down-regulated (≥2-fold; n = 657) or up-regulated (≥2-fold; n = 558) sets. See Figure S4, Figure S5 for the complete list of Z, M, and M/Z genes. Note that 78.2% of down-regulated genes belong to “zygotic genes”, whereas there are more maternal than “zygotic genes” present in the up-regulated set. (C) Fold changes in the expression of the listed zygotic and housekeeping genes in *Stat92E^mat–^* versus wild-type embryos based on the microarray analysis. Average changes and p values (Student's t-Test) are shown. (D) The expression values of a set of 40 “zygotic” and 40 housekeeping genes from the microarray analysis were used for Gene Set Enrichment Analysis (GSEA). See Table S6 for gene names and expression values. Normalized enrichment scores (NESs) and p-values are shown. Note that the “zygotic genes” show highly significant concordant down regulation, whereas the housekeeping genes show insignificant changes.

We found that in *Stat92E^mat–^* embryos, 657 genes were down regulated and 558 genes up-regulated by at least 1.5 fold, compared with wild-type control (Figure 2A). In *Stat92E^mat–^* embryos, genes exhibiting >1.5 fold change in expression constituted 8.9% of all genes (n = 13,615) on the Gene Chip, while the majority (91.1%) of the genes exhibited no significant changes (Figure S2). Consistent with the idea that STAT92E is preferentially required for expression of “zygotic genes”, the vast majority (78.2%) of the down-regulated genes in *Stat92E^mat–^* embryos were “zygotic genes” (Figure 2B, left; Table S4). In contrast, the up-regulated genes contained more maternally expressed than zygotically expressed genes (Figure 2B, right; Table S5). This observation is reminiscent of gene expression profiles of *zld* mutant embryos at the same stage, in which more “zygotic genes” than maternal genes are down-regulated [9]. By comparing the two sets of genes, we found that >50% of the “zygotic genes” that were down-regulated in *zld^mat–^* embryos (67/120) were also down-regulated in *Stat92E^mat–^*embryos, suggesting that these genes might be co-regulated by STAT and Zelda (Table S4).

Consistent with the observed difference in the abundance of STAT-binding sites present in their promoter regions, the 21 zygotic genes (except for *hb*) were all significantly down-regulated, with a 4.3 fold down-regulation on average, whereas the 21 housekeeping genes showed no significant changes in expression, with the exception of DNase II (Figure 2C), in *Stat92E^mat–^* embryos. Similar to *Stat92E^mat–^* embryos, in *zld^mat–^* embryos, many of these 21 zygotic genes were also significantly down-regulated, whereas the housekeeping genes were not significantly changed [9], suggesting that STAT92E and Zelda may both be important for transcription of early zygotic genes. Expression profiling experiments indicate that STAT92E and Zelda do not transcriptionally regulate each other (Liang et al., 2008; this study). We further performed qRT-PCR experiments and found that Zelda mRNA levels were indeed not significantly changed in *Stat92E* loss-of-function or *hop* gain-of-function mutants (Figure S3), suggesting that STAT92E does not indirectly control zygotic gene activation by affecting Zelda levels.

Finally, we tested expanded sets of zygotic and housekeeping genes to include >40 genes in each set (Table S6) using the Gene Set Enrichment Analysis (GSEA) software (http://www.broadinstitute.org/gsea/index.jsp), which is a computational method that determines whether an *a priori* defined set of genes shows statistically significant, concordant differences between two biological states (e.g., mutant versus wild-type) [28]. Indeed, by subjecting our microarray data to GSEA analysis, we found that the “zygotic genes” were highly significantly down regulated (p = 0.00), whereas the housekeeping genes were insignificantly changed (p = 0.44), in *Stat92E^mat–^* embryos when compared with wild-type control (Figure 2D). Thus, similar to Zelda, STAT92E is preferentially required for transcription of “zygotic genes”.

### STAT92E and Zelda co-regulate multiple early “zygotic genes”

To validate our gene profiling results from the microarray studies, we investigated the effects of over-activation and loss of STAT92E on transcript levels of a number of early “zygotic genes”. We chose to examine expression levels of *dpp, Kr, tll*, and *eve*, four early zygotic genes whose promoter regions contain STAT-binding sites and whose expression domains span broad and distinct regions of the early embryo (see below).

**Figure 3:**
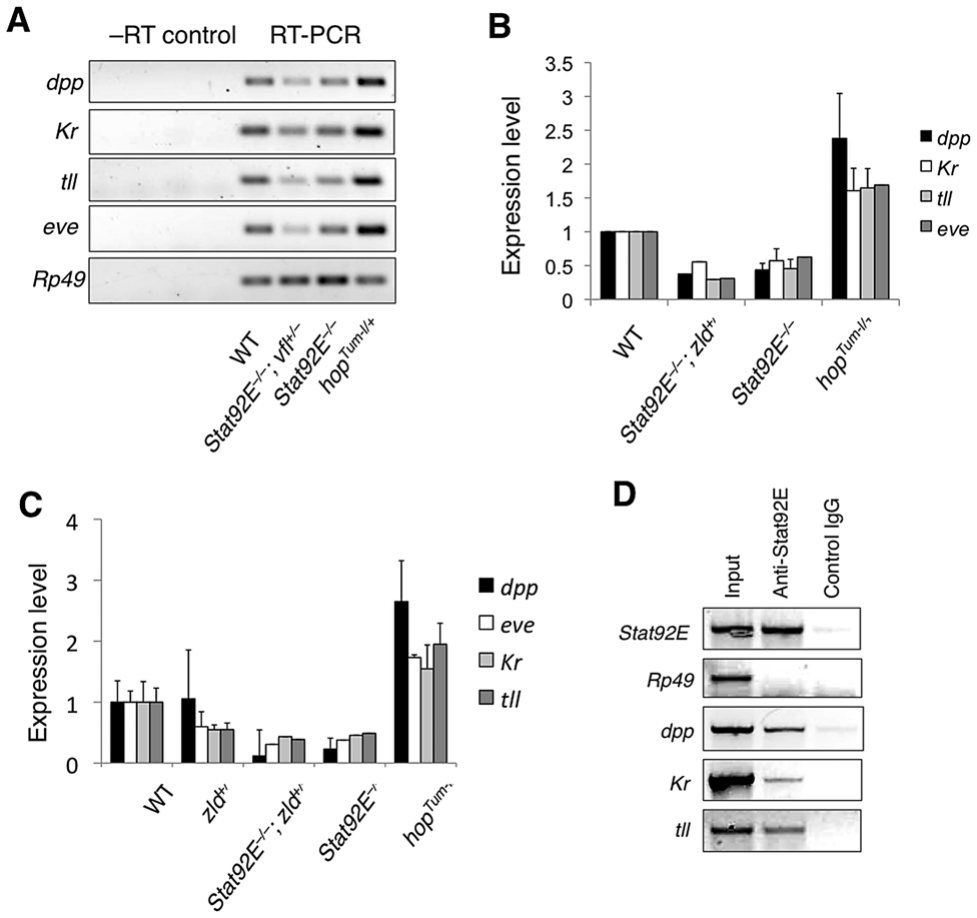
JAK/STAT signaling regulates multiple “zygotic genes.” (A) Total RNA was isolated from staged early embryos (1–2 h after egg laying) of the indicated genotypes, and mRNA levels of *dpp*, *Kr*, *tll*, and *eve* were measured relative to those of *rp49* (control) by semi-quantitative RT-PCR. A representative gel picture is shown. (B) Quantification of the RT-PCR results. Note that the levels of *dpp*, *Kr*, *tll*, and *eve* mRNA were higher in *hop^Tum-l/+^* embryos, lower in *Stat92E^mat–^* embryos, and were further reduced when combined with *zld^+/–^*. (C) Levels of mRNA expression in embryos of indicated genotypes were quantified by real-time PCR. Error bars indicate standard deviation. (D) Early wild-type embryos (1–2 h AEL) were homogenized and used for ChIP experiments with goat anti-STAT92E. An equal amount of goat IgG was used as control. The *Stat92E* promoter was used as a positive control, and the rp49 promoter as a negative control.

We first examined mRNA levels of *dpp*, *Kr*, *tll*, and *eve* in the early embryo (1–2 h after egg laying) using semi-quantitative reverse-transcription polymerase chain reaction (RT-PCR) in *Stat92E* gain- or loss-of-function genetic backgrounds. We found that in *hop*GOF embryos, in which STAT92E is overactivated [29]–[31], mRNA of these four genes were all expressed at significantly higher levels relative to wild-type; whereas in *Stat92E^mat–^* embryos, these four genes were expressed at approximately 50% of the wild-type levels (Figure 3A, 3B). Moreover, reducing the dosage of *zelda* by half in *Stat92E^mat–^* embryos caused further reductions in the transcript levels of *dpp*, *Kr*, *tll*, and *eve* (*zelda^+/–^; Stat92E^mat–^* in Figure 3A, 3B). We examined *zelda^+/–^; Stat92E^mat–^* embryos only, because it was technically not possible to examine embryos lacking both Zelda and Stat92E. We further confirmed the expression results by quantitative real-time PCR (Figure 3C). These results were consistent with the microarray data, which suggested that Stat92E and Zelda may co-regulate transcription of many “zygotic genes”.

We next investigated whether STAT92E binds to the putative STAT-binding sites in the respective promoter regions of *dpp*, *Kr*, and *tll* using chromatin immunoprecipitation (ChIP) experiments with early embryo extracts using anti-STAT92E antisera. Binding of STAT92E to the *eve* enhancer and of Zelda to the TAGteam sequences enriched in “zygotic genes” have been previously shown [9], [19], [21]. Using primers flanking the putative STAT-binding sites in these promoter regions, we detected STAT92E binding to the promoter regions *dpp*, *Kr*, and *tll* (Figure 3D). The results from RT-PCR and ChIP studies were consistent with the bioinformatic and gene profiling studies shown above, suggesting that STAT92E, likely together with Zelda, regulates the transcription of early “zygotic genes” *in vivo*.

### STAT and Zelda cooperate to regulate *dpp* transcriptional regulation

**Figure 4:**
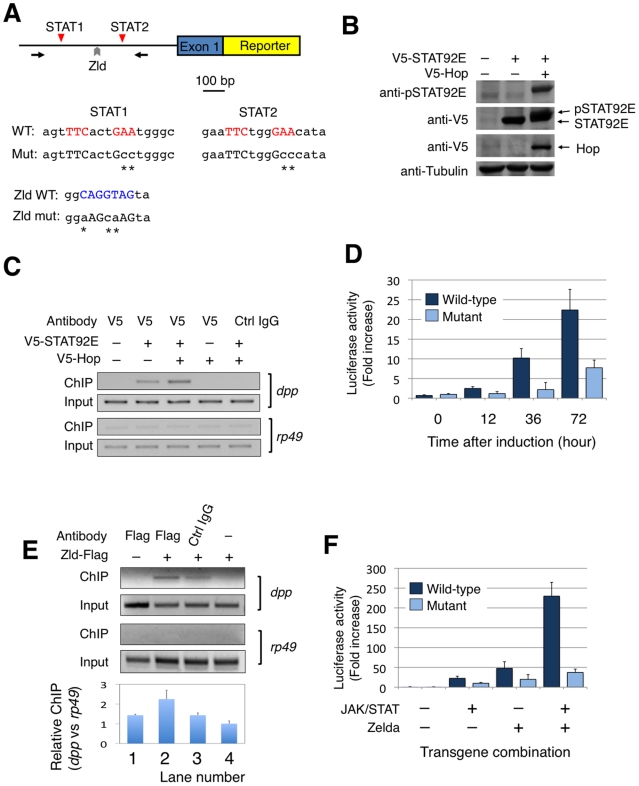
STAT92E and Zelda synergistically regulates *dpp* reporter gene expression in *Drosophila* S2 cells. (A) Schematic representation of the *dpp* reporter constructs with two STAT-binding sites (red triangles; STAT1 and STAT2) and a Zelda binding site (gray arrowhead). Sequence differences between wild-type (WT) and double-mutant (DM) constructs are noted. Arrows represent primers for PCR amplification used in ChIP experiments. Sequences of STAT and Zelda binding sites and the corresponding mutants are shown. (B) *Drosophila* S2 cells were transfected with V5-tagged STAT92E, with or without V5-Hop. Cell lysates were subjected to SDS-PAGE and blotted with indicated antibodies. Note that cotransfection of Hop induces phosphorylation of STAT92E in S2 cells (lane 3). (C, E) Chromatin immunoprecipitation (ChIP) experiments to detect binding of STAT92E and Zelda to *dpp* promoter. S2 cells were transfected with V5-STAT92E with or without Hop, as indicated (C), or with Flag-Zelda (E). Anti-V5 or anti-Flag were used to immunoprecipitate STAT92E or Zelda, respectively. The chromatin in the immunoprecipitates was detected by PCR with primers used in (A). Note that the *dpp* promoter fragment bound to STAT92E is more enriched when Hop is coexpressed (C; lane 3), and that Zelda is enriched in the *dpp* promoter (E; lane 2). Quantification using real-time PCR is shown in lower panel. (D, F) S2 cells were transfected with *dpp^WT^*-luc or *dpp^DM^*-luc, and cotransfected with Hop and STAT (D), or additionally with or without Zelda (F). Hop, STAT, and Zelda were under the control of a metallothionein (MT) promoter. Relative luciferase activity was measured at indicated hours (D) or at 72 hours (F) after induction of the MT promoter by CuSO_4_. Results of three independent experiments are shown. Note that in *dpp^WT^*-luc cells, STAT activation resulted in >20-fold increase in luciferase activity at 72 h after induction (D), Zelda expression resulted in a 50 fold increase in luciferase activity (F, colume 3), and that in presence of activated STAT and co-transfected Zelda, there was >200 fold increase in luciferase activity (F, colume 4).

Having shown that STAT92E regulates expression levels of early “zygotic genes”, and that STAT92E binds to the consensus STAT-binding sites present in the promoter regions of *dpp, Kr,* and *tll*, we next investigated whether these consensus STAT-binding sites are indeed essential for mediating STAT92E transcriptional activation, and whether STAT92E and Zelda cooperate to regulate “zygotic genes”, as it has previously been shown that Zelda is essential for expression of *dpp, Kr, tll*, and *eve*, among others, in the early embryo [9]. We carried out reporter gene assays in *Drosophila* S2 cells (Figure 4A).

We first tested whether activated STAT92E binds to the promoter regions of *dpp*, *Kr*, *tll*, and *eve* in S2 cells as it does in early embryos (see Figure 3C). We transfected a V5-tagged STAT92E into S2 cells and performed ChIP assays. STAT92E activation in S2 cells was achieved by co-expressing Hop, which phosphorylates and activates STAT92E when over-expressed (Figure 4B). By immunoprecipitation with anti-V5 antibody, we found that co-transfection with Hop leads to an enrichment of STAT92E binding to the endogenous *dpp* promoter (Figure 4C, lane 3). Activation of JAK/STAT signaling thus induces a stronger association of STAT92E with the *dpp* promoter, consistent with the idea that STAT92E directly regulates *dpp* expression. However, the same ChIP experiments failed to detect association of STAT92E with the *Kr, tll*, or *eve* promoter in S2 cells, in contrast to the ChIP results in early embryos (see Figure 3C), suggesting that the epigenetic states of these promoter sequences may be different in S2 cells than in early embryos. We thus focused on the *dpp* promoter for reporter gene analysis. To this end, we isolated a 1.3 Kb *dpp* promoter fragment (Figure 4A; Figure S4), which contains the two clustered STAT92E binding sites we had tested in ChIP experiments (see Figure 3C, Figure 4C).

To test whether the STAT-binding sites in the *dpp* promoter are important for JAK/STAT-induced *dpp* expression, we made reporter genes by fusing a wild-type *dpp* promoter fragment (WT), or a mutant version with both STAT-binding sites mutated (DM), with an enhanced yellow fluorescent protein (EYFP), and transfected S2 cells (Figure 4A). In order to activate reporter gene expression, we first treated the cells with H2O2/vanadate (pervanadate), which causes rapid and efficient STAT92E phosphorylation [32], [33] (Figure S5A) and is more efficient than transient transfection of *hop* in activating STAT. We found that, indeed, EYFP was expressed 1.5 hours after pervanadate treatment in S2 cells transfected with the wild-type (WT), but not the double mutant (DM) construct (Figure S5B), indicating that these STAT92E-binding sites are important for phosphorylated STAT92E-induced reporter gene expression.

To more accurately quantify transcription from the *dpp* promoter with or without the two STAT-binding sites, we replaced EYFP with luciferase in the reporter constructs to obtain *dpp^WT^-luc* and *dpp^DM^-luc,* respectively. In addition, we used Hop and STAT92E co-transfection, instead of pervanadate, to ensure specific activation of STAT92E. In the presence of co-transfected Hop and STAT92E, we detected an increase in luciferase activity in S2 cells tranfected with *dpp^WT^-luc* to more than 20 fold when measured 72 hours after transgene expression, and this increase was abolished when *dpp^DM^-luc* was used in the assay, which showed much less pronounced increase (Figure 4D). These results further substantiate our finding that STAT92E-mediated activation of *dpp* requires the two STAT92E binding sites.

It has previously been shown that transcription of *dpp* is significantly down-regulated in the absence of Zelda [9], and that Zelda-binding sites are present in the *dpp* promoter region (Figure 1C; Figure 4A; also see [9]). To test whether Zelda binds to the putative site in the *dpp* reporter gene, we carried out ChIP assays in S2 cells after transfecting a Zelda-Flag plasmid. Indeed, we detected Zelda binding to the *dpp* promoter region using an anti-Flag antibody and ChIP assay (Figure 4E).

We next investigated the role of Zelda in *dpp* transcription using *dpp^WT^-luc* and a mutant promoter fragment with the Zelda-binding site and the two STAT-binding sites mutated (designated as *dpp*™*-luc* as it bears triple mutations; Figure 4A). To evaluate whether Zelda and STAT cooperate in regulating *dpp* transcription, we co-transfected S2 cells with STAT92E (together with Hop to achieve STAT activation) or Zelda, or both STAT92E (with Hop) and Zelda, in the presence of *dpp^WT^-luc* or *dpp*™*-luc*, and carried out luciferase assays. When assayed at 72 h after induction of transgene expression, we found that STAT activation alone induced *dpp^WT^-luc* transcription by 22 fold, and Zelda alone caused upregulation of *dpp^WT^-luc* by 48 fold, whereas in the presence of both Zelda and activated STAT, *dpp^WT^-luc* was up-regulated by 230 fold (Figure 4F). Mutating STAT and Zelda binding sites prevented the dramatic increase in transcription as measured by luciferase activity (Figure 4F). These results suggest that Zelda and STAT have synergistic effects on *dpp^WT^-luc* transcription. Interestingly, an increase in luciferase activity was observed even when binding sites for STAT or Zelda, or both, were mutated, albeit to a much less pronounced level than with the wild-type promoter (Figure 4D, 4F), suggesting that there might be other cryptic binding sites present in the promoter, or that other molecules were activated by over-expressed JAK or Zelda.

The apparent synergy between STAT92E and Zelda could explain the results from the gene profiling experiments. Microarray results show that embryos without STAT92E (in which Zelda presumably remains active) exhibit a 3.1 fold decrease in *dpp* expression (Figure 2B), and that *Zld* mutant embryos (in which presumably STAT92E is still active) have reduced *dpp* expression by 5.7 fold [9]. These data suggest that in the early embryo either Zelda or STAT activation could induce *dpp* transcription to a limited extent, whereas the presence of both Zelda and STAT activation synergistically promote *dpp* transcription.

### STAT92E regulates transcription levels, but not spatial domains, of early zygotic genes

Having shown that STAT92E, possibly acting synergistically with Zelda, is important for expression levels of many early “zygotic genes”, we next investigated whether loss of STAT92E also affects the spatial expression patterns of the early “zygotic genes”. We examined the expression of *dpp*, *Kr*, and *tll* in the early embryo, by *in situ* hybridization, while the effects of *Stat92E* mutation on *eve* expression have previously been documented [19], [21]. These genes are expressed in distinct spatial domains that altogether cover nearly the entire early embryo (see below).

**Figure 5:**
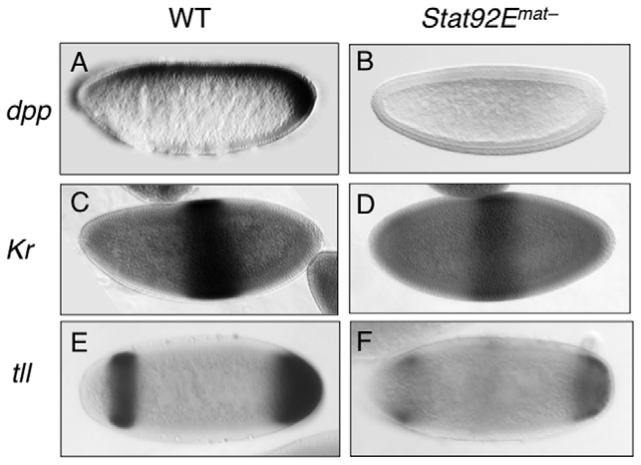
Effects of lacking *Stat92E* on expression of *dpp, Kr*, and *tll* in early embryos. Expression patterns and levels of *dpp, Kr*, and *tll* mRNA or Kr protein (dark stain) were detected by in situ hybridization in precellularization or cellularization stage wild-type (left) and *Stat92E^mat–^* (right) embryos. Stainings were carried out in parallel under identical conditions. Developmental stage was identified by nuclear density, visualized with DAPI stain. Representative embryo images are shown. All embryos are shown with anterior to the left and dorsal up. (A, B) *dpp* expression at the cellularization stage. In wild-type embryos (A), *dpp* mRNA is expressed in the dorsal and posterior regions. In *Stat92E^mat–^* embryos (B), *dpp* expression is much reduced, especially at the posterior pole region. (C, D) Expression of *Kr* mRNA in precellularization stage embryos. Note that in *Stat92E^mat–^* embryos, *Kr* mRNA is expressed in the correct domain but in much reduced levels. (E, F) *tll* mRNA expression at the cellularization stage. Note that in *Stat92E^mat–^* embryos, *tll* is expressed in the correct domains but at lower levels.

The *dpp* expression domain spans nearly the entire A/P axis in the dorsal regions of the early embryo [34]-[37] (Figure 5A). It has been shown that *dpp* transcription in the ventral region is repressed by Dorsal, a Rel family transcription factor [38], and that general transcription factors, such as Zelda and STAT, are responsible for *dpp* expression in the dorsal region ([9]; this study). By employing *in situ* hybridization, we found that compared to wild type, the overall level of *dpp* mRNA is much reduced in *Stat92E*mat– embryos, especially in the posterior pole region (Figure 5B). Moreover, we found that JAK/STAT signaling also regulates *dpp* expression during late embryogenesis (Figure S6). These results are consistent with previous findings in other developmental contexts [39], [40] as well as with the above microarray results and mRNA measurements (Figure 2, Figure 3A–3C).

*Kr* is expressed in the central region of the early embryo [41] (Figure 5C). Other than the maternal morphogens Bcd and Hb, it is not known whether additional factors contribute to *Kr* transcriptional activation. We found that in *Stat92E*mat– embryos, although the overall expression pattern of *Kr* mRNA was little changed, its levels were reduced (Figure 5D), consistent with the microarray and qPCR results.

*tll* is expressed in two domains along the A/P axis-the anterior and posterior pole regions [42] (Figure 5E). The Torso pathway controls *tll* expression by antagonizing its repressors [17], [43]; the identity of transcriptional activators of *tll* remains obscure, although STAT92E has been speculated to contribute to *tll* expression [25]. We have previously shown that STAT92E is essential for the expansion of *tll* expression domains caused by Torso, over-activation, but not for the extent of *tll* spatial expression domains under normal conditions [25]. In addition, we have previously shown that there are two consensus STAT binding sites in the *tll* promoter region that are particularly important for Torso overactionvation-induced ectopic *tll* expression [25]. In light of our finding that STAT92E is important for the expression levels of *dpp*, *Kr*, and *tll*, we reexamined the role of STAT92E in endogenous *tll* expression in *Stat92E^mat–^* and wild-type control embryos by *in situ* hybridization done under identical conditions. We found that, similar to *dpp* and *Kr* mRNA, while the spatial patterns of *tll* expression were not dramatically changed as previously shown [25], the overall levels of *tll* mRNA were significantly reduced in *Stat92E^mat–^* embryos (Figure 5F).

Taken together, the above results indicate that loss of STAT92E led to much reduced expression levels of *dpp*, *Kr*, and *tll*, without affecting their spatial expression domains. Similarly, it has been shown that loss of STAT92E results in reductions, but not complete loss of, *eve* stripe 3 and 5, without affecting the overall spatial expression pattern of *eve* [19], [21]. Thus, STAT92E is likely required for regulating the expression levels of early “zygotic genes”, but not for controlling their spatial patterns.

### Loss of STAT results in multiple defects in embryonic pattern formation

Finally, we investigated the biological consequences of reducing expression levels, without altering spatial domains, of multiple zygotically expressed early genes, as with loss of STAT92E. The correct expression of the early zygotic genes during the MZT is essential for formation of different tissues and body parts at the correct positions, i.e., pattern formation [1]–[3]. Pattern formation in *Drosophila* can be conveniently visualized by examining the exoskeleton (cuticle) morphology of the larva or late embryo [1]–[3].

**Figure 6:**
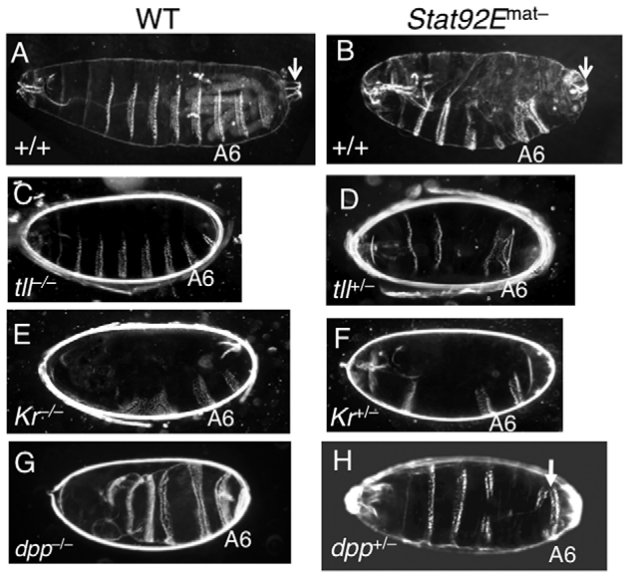
Effects of lacking *Stat92E* on cuticle morphology of embryos. Dark-field images of larval cuticles of different genotypes are shown, with anterior to the left. The position of the sixth abdominal denticle belt is marked as A6. (A) A ventral view of a wild-type larval cuticle, with eight abdominal denticle belts seen in the ventral region, spanning <50% of the body circumference. The arrow points to the Filzkörper, a terminal element. (B) *Stat92E^mat–^* cuticle exhibits defects in central elements (A4, 5) and minor defects in the posterior region, but does not show overt D/V polarity defects. Note that the Filzkörper is present (arrow). (C) *tll^–/–^* embryos are missing posterior terminal structures (A8 and the Filzkörper). (D) *Stat92E^mat–^*; *tll^+/–^* embryos lack posterior terminal structures (A7/8 and the Filzkörper), similar to *tll^–/–^* embryos. (E) *Kr^–/–^* embryos exhibit anterior defects, lacking or having fused A1–5 ventral deticle bands. (F) *Stat92E^mat–^*; *Kr^+/–^* embryos are missing many anterior denticle bands, reminiscent of *Kr^–/–^* embryos. (G) *dpp^–/–^* larvae are ventralized, with denticle belts around the whole body circumference. (H) A portion of *State92E^mat–^*; *dpp^+/–^* larvae are partially ventralized, with posterior denticle belts (usually A6; arrow) extended to the dorsal side, encompassing 80% of the body circumference.

In the wild-type cuticle (Figure 6A), anteroposterior (A/P) polarity is defined by the head skeleton and three thoracic segments in the anterior, followed by the abdominal segments, and the posterior and terminal structure, consisting of the 8^th^ abdominal segment and the Filzkörper (Figure 6A; Arrow). Dorsoventral (D/V) polarity can easily be seen by the positions of the eight abdominal denticle belts, which form in the ventral region, while bare cuticle marks the dorsal region (Figure 6A). Removal of STAT92E from the early embryo resulted in heterogeneous defects, mostly notably along the A/P axis as seen in the larval cuticles, which were missing part or all of A3, A4, A5, and A8 to various degrees (Figure 6B; also see [19], [25]). Thus, loss of STAT92E, which significantly reduces multiple early “zygotic genes” but does not completely eliminate their expression (see Figure 5), leads to heterogeneous patterning defects, consistent with defects in multiple pathways.

To understand the role of STAT92E in individual signaling pathways important for pattern formation, we investigated whether loss of STAT92E could further compromise pattern formation in sensitized genetic backgrounds. To this end, we examined cuticles of *Stat92E^mat–^* embryos that were also heterozygous for *tll*, *Kr,* or *dpp*, and indeed found patterning defects (see below).

The gap gene *tll* is essential for the development of terminal structures [17], [42], and *tll* mutant homozygous embryos do not have A8 and the Filzkörper (Figure 6C). *tll* heterozygous flies, in contrast, are perfectly viable and normal, with cuticles indistinguishable from wild-type controls, according to our own observation. In the absence of STAT92E, however, we found that *tll^+/–^* embryos were missing the terminal structures (A8 and Filzkörper) (Figure 6D). This suggests that without STAT92E, a half dose of *tll^+^* is no longer sufficient for development, consistent with the idea that STAT92E is partially required for *tll* transcriptional output.

*Kr* is required for development of the thoracic and anterior segments, and these segments are missing in *Kr^–/–^* embryos (Figure 6E; also see [44]). *Kr^+/–^* embryos are mostly normal but have subtle anterior defects (Figure S7; also see [44]). In the absence of STAT92E, however, we found that *Kr^+/–^* embryos were missing a large area of the thoracic and anterior regions (Figure 6F), suggesting a haploinsufficiency in the absence of STAT92E, similar to what we observed for *tll*.

The dorsally expressed *dpp* specifies dorsal cell fates and is crucial for the dorsoventral polarity of the embryo, which is reflected in the cuticle by the presence of naked cuticles in the dorsal region and eight abdominal denticle belts in the ventral region (Figure 6A) [37]. Notably, although *dpp* expression was significantly reduced in *Stat92E^mat–^* embryos (Figure 2, Figure 3A–3C, Figure 5B), they did not exhibit gross D/V polarity defects (Figure 6B), suggesting that the residual *dpp* transcripts present in *Stat92E* ^mat–^ embryos are sufficient for specifying dorsal cell fates, or that the reduction in *dpp* expression is compensated for by a reduction in a *dpp* antagonist that is also regulated by STAT92E. Despite the fact that *dpp* is haploinsufficient for viability, *dpp* heterozygous embryos exhibit normal D/V polarity, with clearly discernable ventral denticle belts and bare dorsal cuticles (Figure S7), suggesting that a half dose of *dpp^+^* suffices for D/V patterning (also see [37]. Embryos homozygous for *dpp*, nonetheless, are completely “ventralized,” having denticle belts that extend into the dorsal region to surround the entire D/V axis (Figure 6G; also see [36], [37]). The combination of *Stat92E^mat–^* and *dpp* heterozygosity caused partial ventralization of the embryo; in 13% of *Stat92E^mat–^*; *dpp^+/–^* embryos (n = 11/86), the posterior-most denticle belt extended significantly dorsally to cover approximately 80% of the circumference (Figure 6H, arrow). Similar ventralization defects were never observed in *Stat92E^mat–^* and *dpp^+/–^* embryos (n>500). Thus, in the absence of STAT92E, a half dose of *dpp* is no longer sufficient for dorsoventral patterning, consistent with the notion that STAT92E normally regulates *dpp* expression levels.

In summary, loss of STAT92E caused heterogeneous patterning defects, as revealed by varying cuticle defects, consistent with an insufficiency of multiple pathways. A further reduction in the dosage of genes in different pathways, such as *tll*, *Kr*, and *dpp*, uncovered the role of STAT92E in regulation of specific early zygotic genes important for pattern formation.

## Discussion

We have undertaken a bioinformatics approach to investigating the mechanisms controlling transcription of the zygotic genome that occurs during the MZT, and have identified STAT92E as an important general transcription factor essential for up-regulation of a large number of early “zygotic genes”. We have further investigated the role of STAT92E in controlling transcription of a few representative early zygotic genes, such as *dpp*, *Kr*, and *tll*, that are important for pattern formation and/or cell fate specification in the early embryo. Our studies suggest that STAT92E cooperate with Zelda to control transcription of many “zygotic genes” expressed during the MZT. While STAT mainly regulates transcription levels, but not spatial patterns, of *dpp*, *tll*, and *Kr*, and possibly also other “zygotic genes”, Zelda is essential for both levels and expression patterns of these genes [9].

The transcriptional network that controls the onset of zygotic gene expression during the MZT has remained incompletely understood. It has been proposed that transcription of the zygotic genome depends on the combined input from maternally derived morphogens and general transcription factors. The former are distributed in broad gradients in the early embryo and directly control positional information (e.g., Bicoid, Caudal, and Dorsal), whereas the latter are presumably uniformly distributed regulators that augment the upregulation of a large number of “zygotic genes”. Other than Zelda, which plays a key role as a general regulator of early zygotic expression [9], the identities of these general transcriptional activators have remained largely elusive. It has been shown that combining Dorsal with Zelda- or STAT-binding sites supports transcription in a broad domain in the embryo [10]. The demonstration of STAT92E as another general transcription factor sheds light on the components and mechanisms of the controlling network in the early embryo. Moreover, we have found that STAT92E and Zelda may cooperate to synergistically regulate “zygotic genes”. Our results thus validate the bioinformatics approach as useful in identifying ubiquitously expressed transcription factors that may play redundant roles with other factors and thus might otherwise be difficult to identify.

Our conclusion that STAT92E is important for the levels but not the spatial domains of target gene expression in the early embryo is consistent with several previous reports. It has been shown that in *Stat92E* or *hop* mutant embryos, expression of *eve* stripes 3 and 5 are significantly reduced but not completely abolished [19], [21]. In addition, JAK/STAT activation is required for the maintenance of high levels, but not initiation, of *Sxl* expression during the MZT [45], [46]. Moreover, it has previously been shown that STAT92E is particularly important for Torso^GOF^-induced ectopic *tll* expression but not essential for the spatial domains of *tll* expression in wild-type embryos under normal conditions [25]. On the other hand, Zelda may be important for both levels and spatial patterns of gene expression. This idea is consistent with our finding that Zelda-binding sites are enriched in both promoter and promoter-distal enhancers regions, whereas STAT-binding sites are enriched in promoter regions only. It has been reported that pausing of RNA polymerase II is prominently detected at promoters of highly regulated genes, but not in those of housekeeping genes [47]. In light of our results that STAT and Zelda sites are highly enriched in the early zygotic gene promoters, we suggest that these transcription factors might contribute to chromatin remodeling that favors RNA polymerase II pausing at these promoters.

Finally, the MZT marks the transition from a totipotent state to that of differentiation of the early embryo. As a general transcription factor at this transition, STAT, together with additional factors (such as Zelda [9]), is important for embryonic stem cell differentiation. Further investigation is required to understand the molecular mechanism by which STAT and Zelda [9] cooperate in controlling zygotic transcription in the early *Drosophila* embryo. Moreover, it would be interesting to investigate whether STAT plays similar roles in embryonic stem cell differentiation in other animals.

## Materials and Methods

### Fly stocks and genetics

All crosses were carried out at 25°C on standard cornmeal/agar medium unless otherwise specified. Fly stocks of *hop^Tum-l^*, *Stat92E^6346^*, and *dpp^H46^* were from the Bloomington *Drosophila* Stock Center (Bloomington, IN). To generate *Stat92E* ^mat–^ embryos, *hsp70-flp; FRT^82B^ Stat92E^6346^/TM3* females were crossed to *hsp70-Flp; FRT^82B^ [ovo^D1^, w^+^]/TM3* males. Their 3rd instar larval progeny were heat-shocked at 37°C for 2 hrs daily for 3–4 days, and resulting adult females of the genotype *hsp70-flp; FRT^82B^ Stat92E^6346^/FRT^82B^ [ovo^D1^, w^+^]* were used to produce embryos that lack maternal *Stat92E* gene products, as described in the dominant female-sterile “germline clone” technique [48].

### Bioinformatic analyses

The following rules were used for assigning a score to known or putative activators of each of the “zygotic genes”. We placed top importance on genetically demonstrated activation during early embryogenesis, with such an activator receiving an activation score of 10. For instance, Torso was assigned a score of 10 as an activator of *tll* transcription based on the reports that *tll* is not expressed in *torso* loss-of-function mutants and is overexpressed in *torso* gain-of-function mutants [17], [49]. Activators identified by biochemical/promoter studies in early embryos or by genetic studies at other developmental stages were assigned a score of 5. Lower scores were assigned to other less stringent evidence of interaction, such as unconfirmed genetic screen results (5), in vitro biochemical assays (2), or bioinformatics studies (1) (Table S1).

Databases and programs used in this study:

Flybase (http://flybase.org); PubMed (http://www.ncbi.nlm.nih.gov); RedFly (http://redfly.ccr.buffalo.edu/); FlyEnhancer (http://genomeenhancer.org/fly).

### DNA constructs and plasmids

The *dpp* promoter used in this study was a 1.3 kb genomic DNA fragment including the upstream regulatory sequences and the non-coding exon 1 of the of *dpp* transcript A (Figure S2). This genomic region has previously been shown to be the core promoter of *dpp* [38]. Standard cloning was used to generate transcription fusions between the *dpp* promoter and cDNAs of reporter genes, such as enhanced yellow fluorescent protein (EYFP) and luciferase. Mutagenesis of two STAT92E binding sites within the *dpp* promoter was done by PCR, and was verified by sequencing. V5-Hop and V5-STAT92E are gifts from S.X. Hou [50].

### Examination of embryos

Cuticle preparations were performed according to a standard protocol with minor modifications. Embryos were dechorionated with 50% Clorox, washed extensively with 0.1% Triton, mounted in Hoyer's, and photographed using dark-field optics. *In situ* hybridization for detecting *dpp*, *Kr*, and *tll* mRNA was performed according to a standard protocol using digoxigenin-incorporated antisense RNA probes made from *dpp, Kr*, and *tll* cDNA, respectively, according to the supplier's protocol. A standard protocol was used for antibody staining of embryos, and a biotinylated secondary antibody and the Vectastain ABC kit (Vector Laboratories, Inc.) were used according to the manufacturer's instructions. Stained embryos were mounted in DAPI-containing mounting medium for accurate staging, when necessary. Mounted embryos were photographed using Normaski optics on a Zeiss Axioscope and images were analyzed using Photoshop or ImageJ software.

### Microarray, semi-quantitative RT-PCR, and quantitative real-time PCR

Total RNA was isolated from embryos (from flies raised at 25°C) collected at 1–2 h after egg laying (corresponding to nuclear division cycles 8–14) using trizol (Invitrogen) or the RNeasy Kit (QIAGEN) according to the manufacturer's instructions. RNA quality was assessed using the Agilent 2100 Bioanalyzer and the RNA 6000 Nano kit (Agilent Technologies Inc., Palo Alto, CA).

For RT-PCR analysis, first strand complementary DNA (cDNA) was generated from 5 µg of purified total RNA using Superscript III reverse transcriptase (Invitrogen) and oligo(dT)12–18 in 50 µl total reaction volume. The cDNA (at 1∶100 dilution) was used as template for either semi-quantitative PCR reactions or real time PCR analysis using SYBR green based detection on a BioRad iCycler. Reactions were carried out in triplicate, and melting curves were examined to ensure single products. Results were quantified using the “delta-delta Ct” method to normalize to *rp49* transcript levels and to control genotypes. Data shown are averages and standard deviations from at least three independent experiments. The following primer pairs were used.

*rp49*: TCCTACCAGCTTCAAGATGAC, CACGTTGTGCACCAGGAACT.

*dpp*: AATCAATCTTCGTGGAGGAGCCGA, TTGGTGTCCAACAGCAGATAGCTC.

*eve*: TGCACGGATACCGAACCTACAACA, GTTCTGGAACCACACCTTGATCGT.

*Kr*: CAAGACGCACAAACGCGAACCTTA, TTGACGGTTTGCAGCCAGAAGTTG.

*tll*: AATACAACAGCGTGCGTCTTTCGC, ACATTGGTTCCTGTGCGTCTTGTC.

For microarray analysis, 200 ng of total RNA was used to prepare biotin-labeled RNA using Ambion MessageAmp Premier RNA Amplification Kit (Applied Biosystems, Foster City, CA). Briefly, the first strand of cDNA was synthesized using ArrayScript reverse transcriptase and an oligo(dT) primer bearing a T7 promoter. Then DNA polymerase I was used (in the presence of *E. coli* RNase H and DNA ligase) to convert single-stranded cDNA into a double-stranded DNA (dsDNA). The dsDNA was then used as a template for in vitro transcription in a reaction containing biotin-labeled UTP and T7 RNA Polymerase to generate biotin-labeled antisense RNA (aRNA). Twenty µg of labeled aRNA was fragmented and fifteen µg of the fragmented aRNA was hybridized to Affymetrix *Drosophila* Genome 2.0 Array Chips according to the manufacterer's Manual (Affymetrix, Santa Clara, CA). Array Chips were stained with streptavidin-phycoerythrin, followed by an antibody solution (anti-streptavidin) and a second streptavidin-phycoerythrin solution, performed by a GeneChip Fluidics Station 450.

The Array Chips were then scanned with the Affymetrix GeneChip Scanner 3000. The microarray image data were converted to numerical data with Genespring software (Agilent Technologies Inc., Palo Alto, CA) and normalized using the recommended defaults. The signals from 11 perfect matched oligonucleotides for a specific gene and 11 mis-matched oligonucleotides were used to make comparisons of signals. Genes were identified as present when the present (P) assignment was significant (p<0.05).

The Gene Set Enrichment Analysis (GSEA) online software (http://www.broadinstitute.org/gsea) was used to determine whether the predetermined gene sets (e.g., zygotic versus housekeeping; see Figure S6) show statistically significant, concordant differences between wild-type and *Stat92E^mat–^* embryos.

### Antibodies and cell culture

Primary antibodies used in this study include mouse anti-V5 (Invitrogen; 1∶500 for Western blots), Rabbit anti-V5 (QED; 1∶200 for immunoprecipitation), goat anti-STAT92E (Santa Cruz; Cat# sc-15708; affinity-purified against the N-terminus of STAT92E; 1∶200), rabbit anti-Kr (1∶5000; a kind gift from C. Rushlow), and anti-phospho-STAT92E (Cell Signaling Technology; 1∶1000). Common secondary antibodies were used in whole-mount immunostaining or Western blots.

*Drosophila* Schneider L2 (S2) cells were cultured at 25°C in *Drosophila* Serum-Free Medium (SFM; Invitrogen) supplemented with 10% Fetal Bovine Serum (FBS; Invitrogen) and 0.5x Antibiotic-Antimycotic (Invitrogen). Cells were cultured at 2.5×10^6^/ml prior to transfection. Transfections were performed with FuGene 6 (Roche) according to the manufacturer's instructions. Cu_2_SO_4_ (Sigma) was added to the medium at a final concentration of 0.5 mM 16 hours after transfection, and cells were harvested 48 hours after induction. To stimulate JAK/STAT activation in S2 cells, 2 mM H_2_O_2_ and 1 mM sodium vanadate (pervanadate) were added to the medium and cells were harvested at desired times after treatment. Treated S2 cells were harvested in cell lysis buffer (from Cell Signaling Tech.) for Western blotting or ChIP experiments.

### Chromatin immunoprecipitation (ChIP)

ChIP experiments were carried out according to standard protocols with the following modifications. 200 µl of early embryos (1–2 h AEL) or 1×107 S2 cells were treated with 1% formaldehyde at room temperature for 20 min (embryos) or 10 min (cells) to crosslink protein with chromatin/genomic DNA. Embryos or cells were homogenized and lysed in 300 µl of RIPA lysis buffer with 2 mM EDTA and protease inhibitors on ice. The lysate was sonicated to shear the genomic DNA to lengths between 500 and 1000 bp. An aliquot (50 µl) of sonicated sample was saved as the input control. 5 µg goat anti-STAT92E (Santa Cruz, CA) or rabbit anti-V5 antibodies were added to 200 µl experimental samples in RIPA buffer with 2 mM EDTA and protease inhibitors, and the mixture was incubated overnight at 4°C with rotation. Protein G beads (Sigma), pre-blocked with sonicated salmon sperm DNA (Stratagene), were added to precipitate the antibody-bound chromatin and the precipitate was washed extensively. After reversing the crosslink, DNA was recovered by using a Qiagen PCR purification kit and quantified by PCR. The following forward and reverse primers (flanking two STAT-binding sites in the respective promoter regions) were used for PCR reactions.

*dpp*: AATTCCGGATAGCGCCTGG, AAAGATGGCACACGCTGGG.

*Kr:* CATGCGTTTGCATACTGGAG, CTATTCGAATCGCCCTTGTC.

*tll:* AGTGCTTTGAGGTCGGAATG, AAGAAACCGTGGTGTCCTTG.

*Stat92E:* TGACTGCCCGCTTTTATACC, CAAACGGCGGTCAATAGTTT.

## Supporting Information

Figure S1 Distribution of STAT and Zelda-binding sites in promoter-distal enhances. Dashed horizontal lines represent genomic DNA sequences surrounding the promoter regions from-4000 bp to +1 (transcription start site) of the indicated early zygotic genes. Known enhancers (excluding those localized in the-4000 to +1 bp promoter regions are indicated by solid horizontal line, with base-pair position relative to transcription start site indicated. // denotes discontinuous sequences. Enhancer information was compiled from FlyBase and the references therein. Red triangles represent consensus STAT92E binding sites (TTCnnnGAA). Gray arrowheads indicate the positions of Zelda-binding consensus sequences (CAGGTAG).
(GIF)

Figure S2 Gene expression profile of *Stat92E* mutant versus wild-type control. Total RNA isolated from 1–2 h wild-type and *Stat92E^mat–^* embryos were subjected to microarray analysis. The expression level of each gene is plotted as the log of the average ratio of fluorescent intensity on the *Stat92E^mat–^* chip to the wild-type control chip. Note that expression levels of the majority of the genes were not changed (centered at 0). The gene number is from the Agilent microarray chip.
(GIF)

Figure S3 *Zelda* transcription levels in different genetic backgrounds. Total RNA was isolated from staged early embryos (1–2 h after egg laying) of the indicated genotypes, and mRNA levels of *zelda* and *rp49* (control) were measured by real-time RT-PCR. Zelda expression levels are shown as relative to rp49 and normalized to wild-type control. Three independent experiments were averaged. Error bars are standard deviations.
(GIF)

Figure S4 *dpp* genomic region and enhancer sequence. (A) Horizontal line indicates the genomic region of *dpp*, which can be divided into three regions based on functional requirements for *dpp*, as indicated on top. Transcript A of *dpp* is shown; filled boxes indicate coding, and gray boxes non-coding, regions. The position of the 1.3 kb promoter region is shown. (B) A 500 bp sequence within the 1.3 kb promoter is shown. STAT92E consensus sites are marked in blue, Zelda site in red. (C) Comparison of the putative STAT92E and Zelda binding sites in the *dpp* promoter with the consensus sequences is shown. Numbers indicate positions of the sites relative to the start of *dpp* transcript A.
(GIF)

Figure S5 STAT activation induces *dpp* reporter gene expression S2 cells. (A) *Drosophila* S2 cells were transfected with STAT92E-V5 and were stimulated with H_2_O_2_/vanadate. Cells were lysed 30 min after stimulation and were subjected to SDS-PAGE. STAT92E phosphorylation was detected with anti-pSTAT92E, and transfected STAT92E was detected with anti-V5. Anti-Tubulin was used as a loading control. (B) S2 cells were transfected with *dpp^WT^*-EGFP or *dpp^DM^*-EGFP, and pervanadate treatment was used to activate endogenous STAT92E. EGFP was imaged by confocal microscopy at the same settings for both constructs at different time points after stimulation. Note that EGFP expression in *dpp^WT^*-EGFP transfected cells was detected 1.5 h following pervanadate treatment, but not in *dpp^DM^*-EGFP transfected cells. Right panels are higher magnifications of the white square in the left panel.
(GIF)

Figure S6 JAK/STAT signaling regulates *dpp* expression in late stage embryos. (A, B) In *hop^GOF/+^* embryos, *dpp* expression is increased, but remains excluded from the ventral-most region (arrow in A). The cuticle morphology appears mostly normal (B). (C, E, G) *dpp* expression in parasegment 7 (ps7; arrow) of stage 14 embryos. (D, F, H) Stage 16 embryos were stained with anti-Crumb to reveal the gut epithelia. (C, D) In wild-type embryos, *dpp* is expressed bilaterally at ps7 and other tissues (not marked), as has previously been shown [51]. The midgut exhibits constrictions (folding), which depend on the correct ps7 *dpp* expression, as has previously been shown [52], [53]. (E, F) In *Hop^GOF^* embryos, *dpp* expression at ps7 is increased in intensity, although the midgut appears mostly normal in morphology, albeit slightly over-constricted compared to wild type. (G, H) In *Stat92E^mat–^* embryos, *dpp* expression at ps7 is much reduced or undetectable. The midgut lacks constriction.
(GIF)

Figure S7 Larval cuticle morphology. (A) A wild-type larval cuticle, with eight abdominal denticle belts seen in the ventral region. (B) A *Kr^1/+^* cuticle showing minor anterior defects such as a weakened A2 (arrowhead). (C) *dpp^+/–^* larvae exhibit mostly normal cuticle morphology, with correct D/V polarity, albeit occasional denticle defects.
(GIF)

Table S1 Early zygotic genes and their activators. Activators are based on published literature and may not be transcription factors or directly act on target genes.
(XLSX)

Table S2 Activators of zygotic transcription and their activation score.
(XLSX)

Table S3 Housekeeping genes and STAT-binding site distribution in their promoters.
(XLS)

Table S4 Genes down-regulated in *Stat92E^mat–^* early embryos.
(XLS)

Table S5 Genes up-regulated in *Stat92E^mat–^* early embryos.
(XLS)

Table S6 Zygotic and housekeeping gene sets.
(XLSX)
