# Supplementary figures and images for: STAT Is an Essential Activator of the Zygotic Genome in the Early Drosophila Embryo
Source: PLoS Genet. 2011 May 26;7(5):e1002086. doi: 10.1371/journal.pgen.1002086 (PMC3102735; doi:10.1371/journal.pgen.1002086)

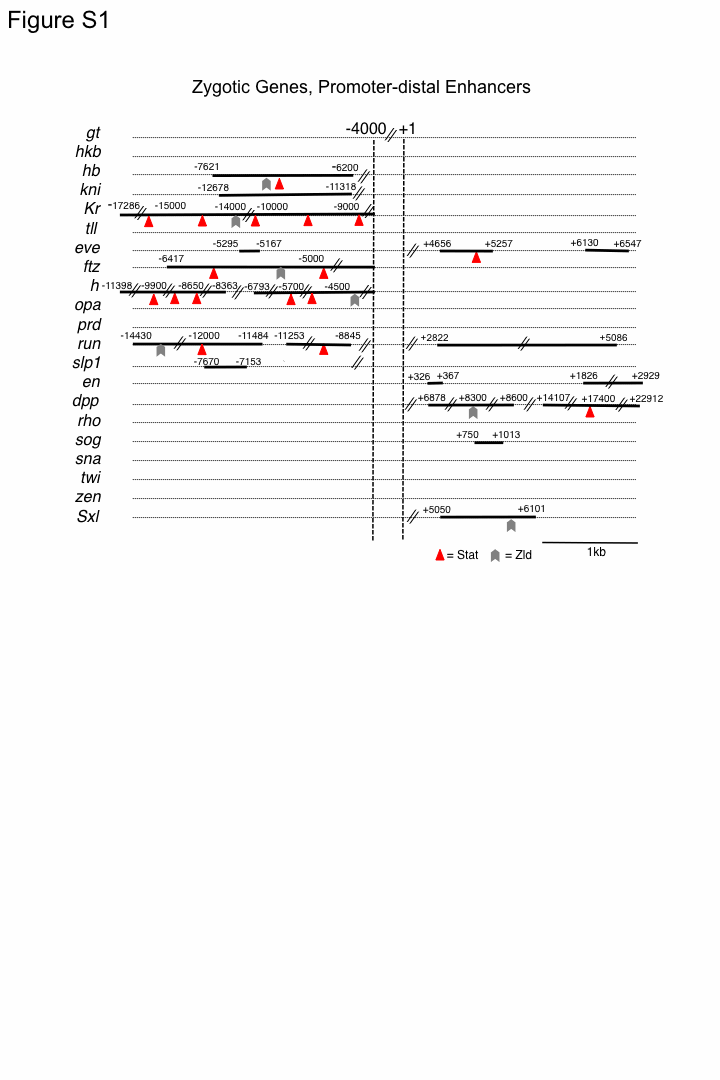

Supplement: Figure S1 — Distribution of STAT and Zelda-binding sites in promoter-distal enhances. Dashed horizontal lines represent genomic DNA sequences surrounding the promoter regions from-4000 bp to +1 (transcription start site) of the indicated early zygotic genes. Known enhancers (excluding those localized in the-4000 to +1 bp promoter regions are indicated by solid horizontal line, with base-pair position relative to transcription start site indicated. // denotes discontinuous sequences. Enhancer information was compiled from FlyBase and the references therein. Red triangles represent consensus STAT92E binding sites (TTCnnnGAA). Gray arrowheads indicate the positions of Zelda-binding consensus sequences (CAGGTAG). (GIF) [file pgen.1002086.s001.gif]

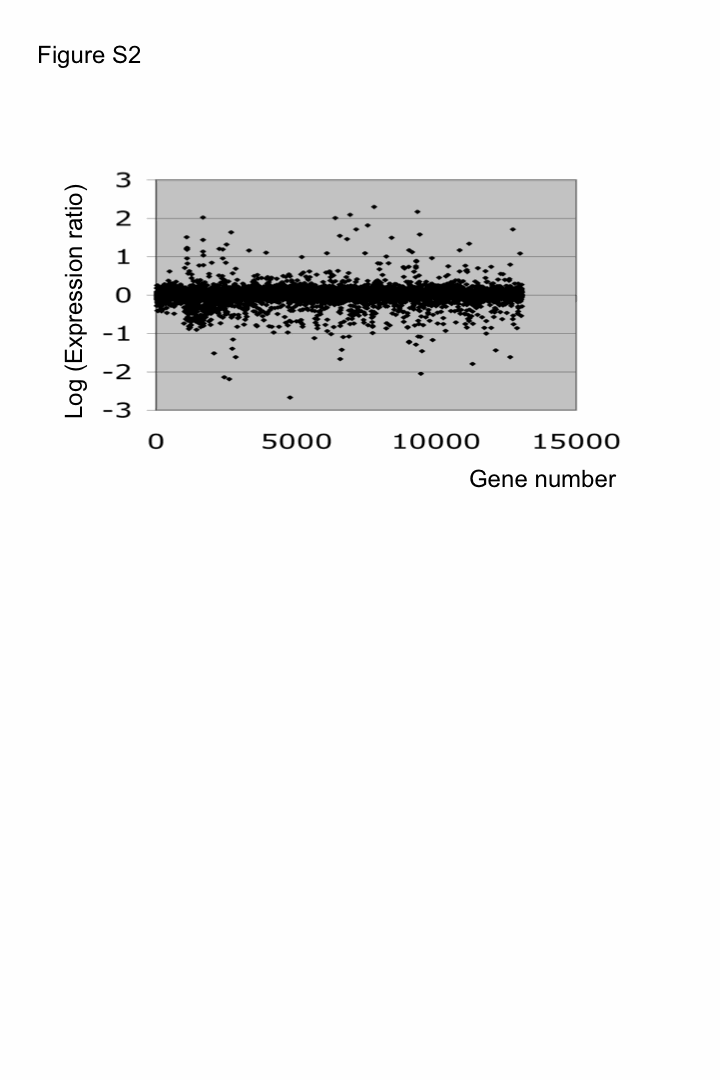

Supplement: Figure S2 — Gene expression profile of Stat92E mutant versus wild-type control. Total RNA isolated from 1–2 h wild-type and Stat92Emat– embryos were subjected to microarray analysis. The expression level of each gene is plotted as the log of the average ratio of fluorescent intensity on the Stat92Emat– chip to the wild-type control chip. Note that expression levels of the majority of the genes were not changed (centered at 0). The gene number is from the Agilent microarray chip. (GIF) [file pgen.1002086.s002.gif]

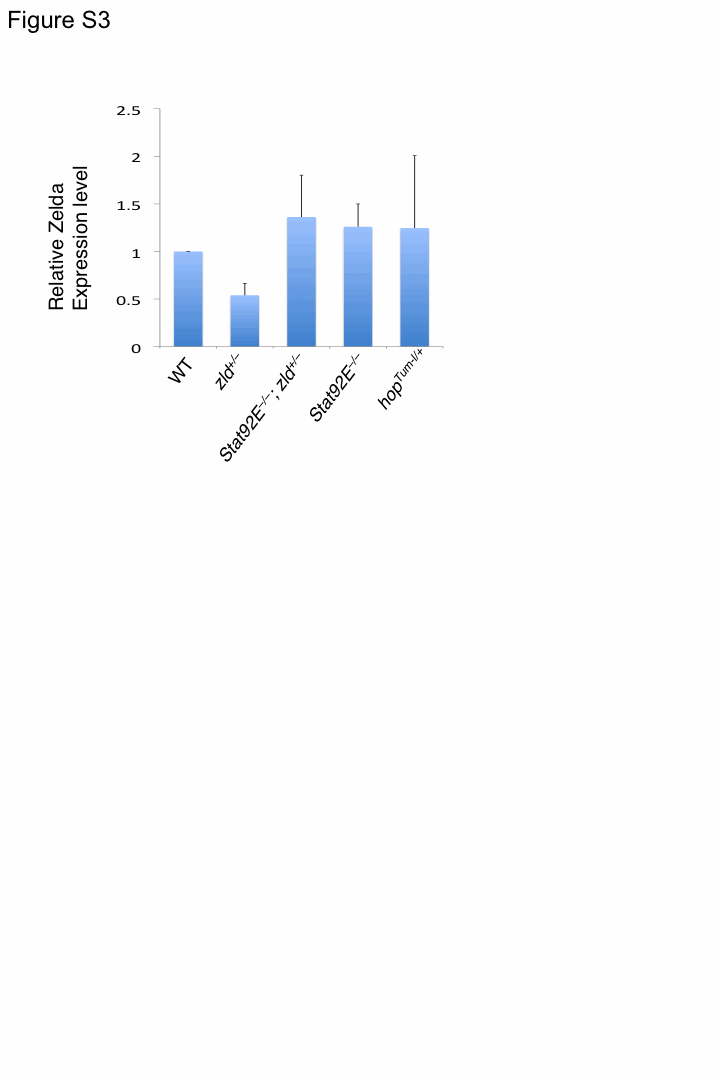

Supplement: Figure S3 — Zelda transcription levels in different genetic backgrounds. Total RNA was isolated from staged early embryos (1–2 h after egg laying) of the indicated genotypes, and mRNA levels of zelda and rp49 (control) were measured by real-time RT-PCR. Zelda expression levels are shown as relative to rp49 and normalized to wild-type control. Three independent experiments were averaged. Error bars are standard deviations. (GIF) [file pgen.1002086.s003.gif]

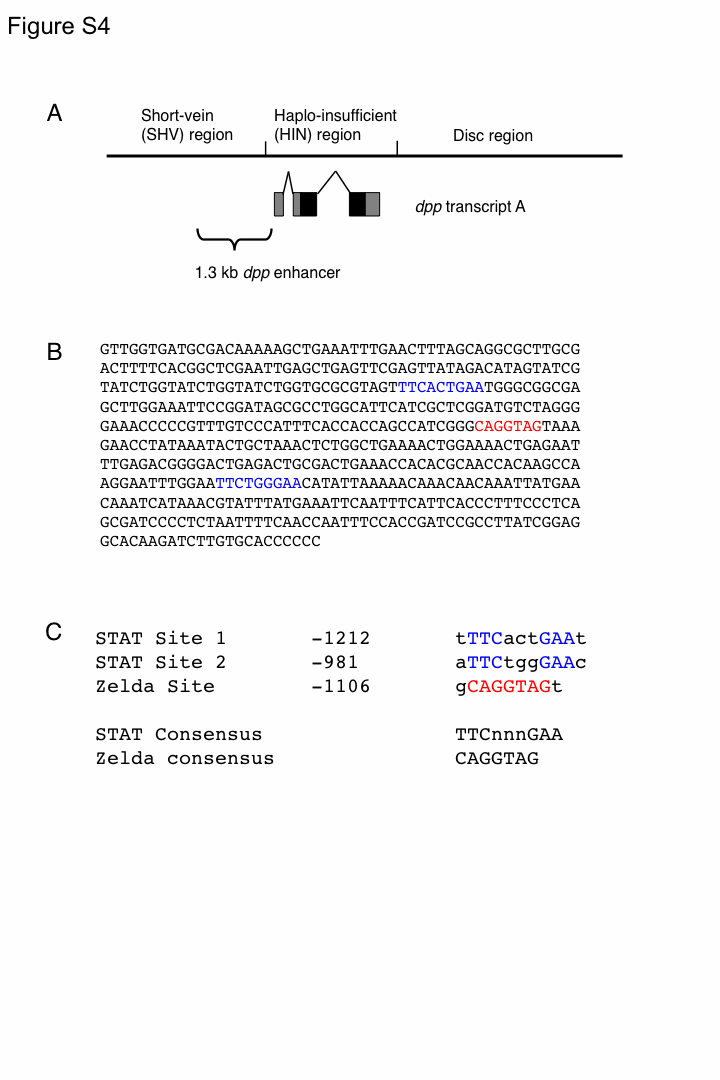

Supplement: Figure S4 — dpp genomic region and enhancer sequence. (A) Horizontal line indicates the genomic region of dpp, which can be divided into three regions based on functional requirements for dpp, as indicated on top. Transcript A of dpp is shown; filled boxes indicate coding, and gray boxes non-coding, regions. The position of the 1.3 kb promoter region is shown. (B) A 500 bp sequence within the 1.3 kb promoter is shown. STAT92E consensus sites are marked in blue, Zelda site in red. (C) Comparison of the putative STAT92E and Zelda binding sites in the dpp promoter with the consensus sequences is shown. Numbers indicate positions of the sites relative to the start of dpp transcript A. (GIF) [file pgen.1002086.s004.gif]

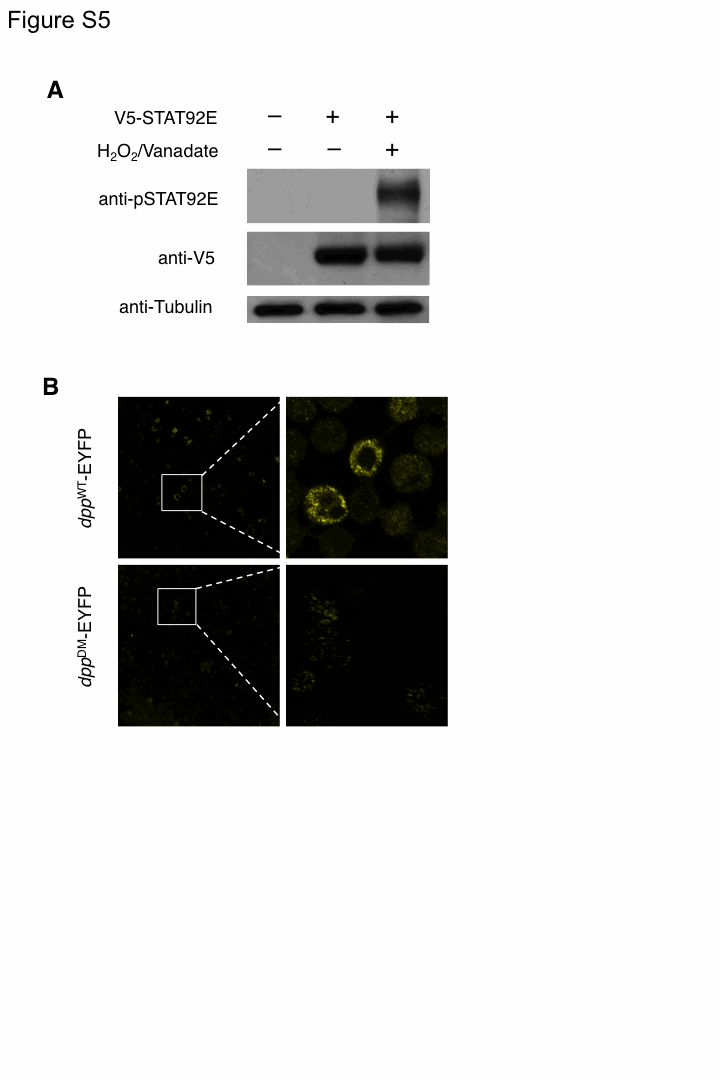

Supplement: Figure S5 — STAT activation induces dpp reporter gene expression S2 cells. (A) Drosophila S2 cells were transfected with STAT92E-V5 and were stimulated with H2O2/vanadate. Cells were lysed 30 min after stimulation and were subjected to SDS-PAGE. STAT92E phosphorylation was detected with anti-pSTAT92E, and transfected STAT92E was detected with anti-V5. Anti-Tubulin was used as a loading control. (B) S2 cells were transfected with dppWT-EGFP or dppDM-EGFP, and pervanadate treatment was used to activate endogenous STAT92E. EGFP was imaged by confocal microscopy at the same settings for both constructs at different time points after stimulation. Note that EGFP expression in dppWT-EGFP transfected cells was detected 1.5 h following pervanadate treatment, but not in dppDM-EGFP transfected cells. Right panels are higher magnifications of the white square in the left panel. (GIF) [file pgen.1002086.s005.gif]

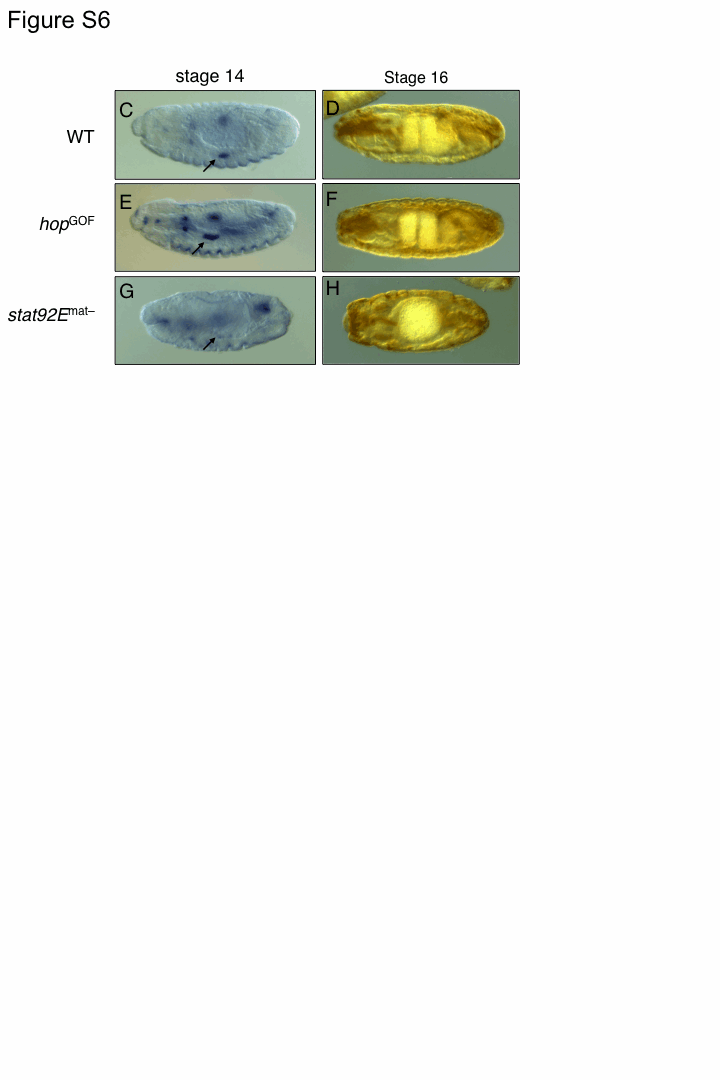

Supplement: Figure S6 — JAK/STAT signaling regulates dpp expression in late stage embryos. (A, B) In hopGOF/+ embryos, dpp expression is increased, but remains excluded from the ventral-most region (arrow in A). The cuticle morphology appears mostly normal (B). (C, E, G) dpp expression in parasegment 7 (ps7; arrow) of stage 14 embryos. (D, F, H) Stage 16 embryos were stained with anti-Crumb to reveal the gut epithelia. (C, D) In wild-type embryos, dpp is expressed bilaterally at ps7 and other tissues (not marked), as has previously been shown [51]. The midgut exhibits constrictions (folding), which depend on the correct ps7 dpp expression, as has previously been shown [52], [53]. (E, F) In HopGOF embryos, dpp expression at ps7 is increased in intensity, although the midgut appears mostly normal in morphology, albeit slightly over-constricted compared to wild type. (G, H) In Stat92Emat– embryos, dpp expression at ps7 is much reduced or undetectable. The midgut lacks constriction. (GIF) [file pgen.1002086.s006.gif]

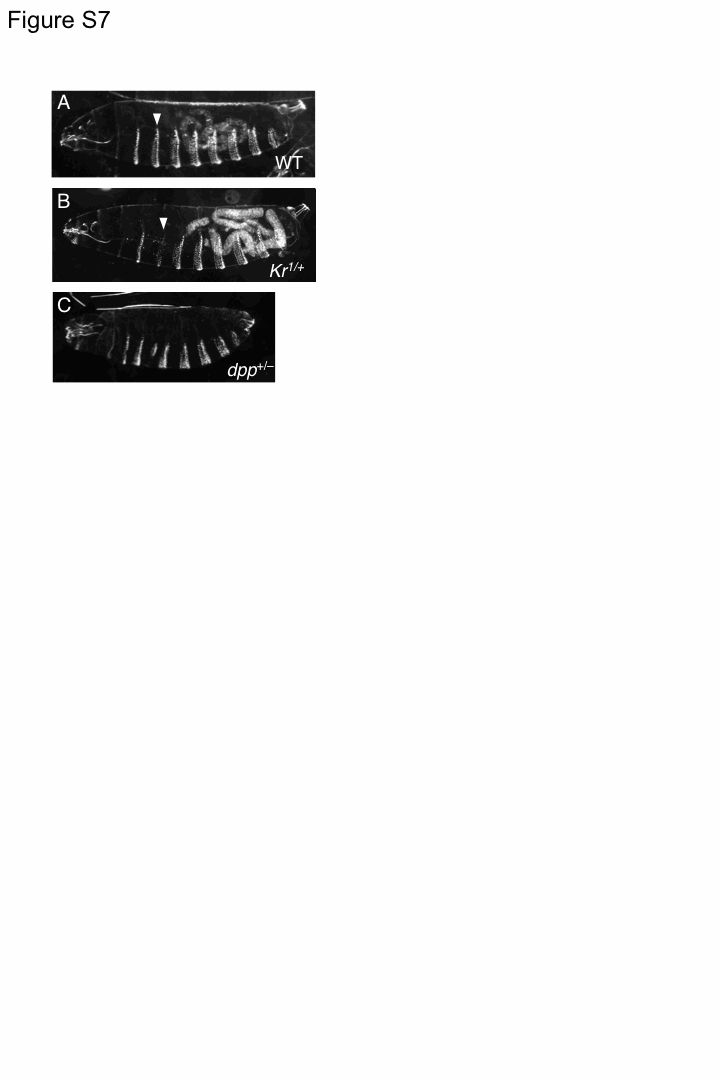

Supplement: Figure S7 — Larval cuticle morphology. (A) A wild-type larval cuticle, with eight abdominal denticle belts seen in the ventral region. (B) A Kr1/+ cuticle showing minor anterior defects such as a weakened A2 (arrowhead). (C) dpp+/– larvae exhibit mostly normal cuticle morphology, with correct D/V polarity, albeit occasional denticle defects. (GIF) [file pgen.1002086.s007.gif]
